# Polyoxyethylene Group-Dependent Surface Properties and Aggregation Behavior in Oleyl-Based Sulfosuccinate Systems

**DOI:** 10.3390/molecules30112321

**Published:** 2025-05-26

**Authors:** Ping Li, Zhengwei Zhang, Jie Chai, Yuan Liu, Siqi Han, Peixin Bai

**Affiliations:** Department of Chemistry and Chemical Engineering, Jinzhong University, Jinzhong 030619, China; tianpinger_2013@126.com (Z.Z.); tian16898@sina.com (J.C.); liuy@jzxy.edu.cn (Y.L.); hansq@jzxy.edu.cn (S.H.); baipx@jzxy.edu.cn (P.B.)

**Keywords:** oleyl-based sulfosuccinates, dynamic surface tension, vesicle, foam stability, wetting ability, liquid crystal emulsion

## Abstract

Three oleyl-based sulfosuccinates with different polyoxyethylene (EO) chain length (MS-OE_n_, where *n* = 3, 5, 7) were synthesized, and their structure were confirmed using FT-IR and ¹H NMR analyses. The surfactant’s adsorption properties, aggregation behavior and practical performance were systematically investigated. Equilibrium surface tension measurements elucidated the surface adsorption properties such as critical micelle concentration (cmc) values and the corresponding surface tensions at cmc (*γ*_cmc_). Dynamic surface tension analysis indicated slower adsorption kinetics for surfactants with longer EO chains. Aggregation studies demonstrated that MS-OE_3_ formed vesicles, whereas no such vesicular structures were observed in the aqueous solutions of MS-OE_5_ and MS-OE_7_ at equivalent concentrations. Further, it was observed that foam stability decreased with an increase in EO units, while MS-OE_3_ exhibited the best wetting ability. Notably, the liquid crystal emulsion formulated with MS-OE_7_ demonstrated exceptional long-term stability.

## 1. Introduction

Sulfosuccinates, a notable class of anionic surfactants, are highly valued for their unique combination of good emulsifying, wetting, and permeability properties. These surfactants are extensively applied in daily chemical industry, dyeing, enhanced oil recovery, leather, and other fields [1,2,3]. The early developed sulfosuccinate was mainly sodium bis(2-ethylhexyl) sulfosuccinate (also known as AOT), and it was widely used as penetrating agent and dispersant due to its remarkable interfacial properties [4]. However, the solubility of AOT in water is quite low. At 30 °C it is about 1.8% (*w*/*w*) [5]. In the mid-1980s, there were significant breakthroughs in both the quantity and variety of these surfactants. Among them, disodium salt of sulfosuccinate monoester was generally used in personal care products including shampoos, cosmetics, facial cleansers, and bubble baths due to its mildness and good biodegradability [6,7], and which was the chief drivers of the sulfosuccinate market growth. In 2023, the global sulfosuccinate market was valued at about $446.2 M.

The typical process for synthesizing sulfosuccinate—based surfactants entails the esterification of maleic anhydride (MAH) with long-chain alcohols to generate the corresponding monoester of maleic acid. Following this, sodium sulfite is introduced into the monoester to yield the final product [8]. Sulfosuccinates with virous structures have been produced and their properties are well established. Richard Wibel [9] synthesized monoalkyl and dialkyl sulfosuccinates with linear and branched hydrophobic tails to gain systematic insights into which hydrophobic tail is most beneficial for hydrophobic ion pairing, and the findings indicated that branched and unsaturated alkyl tails were preferable to saturated linear ones. Satoru Okamura [10] investigated the influence of hydrophobic tail structure on interfacial tension at a hydrophobic solid-water interface using AOT, dioctyl sulfosuccinate sodium salt (Oct), and dihexyl sulfosuccinate sodium salt (Hex). The study demonstrated that a 20 mM AOT aqueous solution could spontaneously detach a triolein droplet from the hydrophobic solid surface in water. According to a report by Linyong Son et al. [11], alkyl polyoxyethylene sulfosuccinate (APS) surfactants exhibited superior tolerance to cations because of the presence of polyoxyethylene (EO) groups. In comparison with octylphenol polyoxyethylene (10) ether sulfosuccinate (OPS), disodium cetyl polyoxyethylene (25) ether sulfosuccinate (CPS) was found to be more effective in the preparation of polystyrene multiporous spheres. This enhanced effectiveness was primarily attributed to the longer EO chain length in CPS. While sulfosuccinates with saturated alkyl chains containing fewer than 12 carbons have been extensively studied, research on sulfosuccinates with high-carbon unsaturated alkyl chains remains relatively limited.

Oleochemicals, derived from renewable resources, are increasingly being utilized as eco-friendly alternatives to petroleum-based products, driven by growing environmental and health concerns associated with conventional surfactants. With the recent volatility in coconut oil prices, oleyl alcohol, a long-chain unsaturated alcohol derived from natural fats and oils [12,13], would regain prominence as a cost-effective and sustainable raw material.

**Scheme 1:**
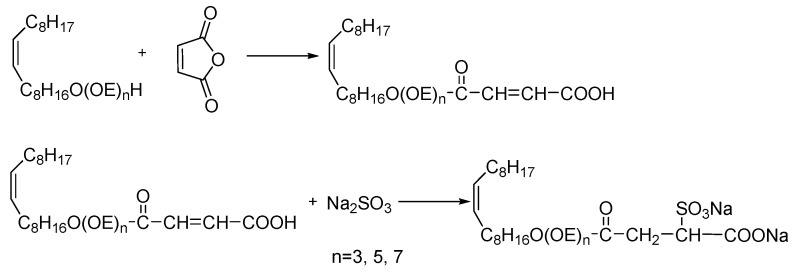
Synthetic route for MS-OE_n_ (*n* = 3, 5, 7).

In this paper, three oleyl-based sulfosuccinates (MS-OE_n_, *n* = 3, 5, 7) were synthesized by esterification of MAH and oleyl alcohol polyoxyethylene ether (OE_n_ *n* = 3, 5, 7) and then sulfonation with sodium sulphite. The synthetic route was depicted in Scheme 1. The adsorption and aggregation behavior of MS-OE_n_ were systematically investigated through static and dynamic surface tension measurements, complemented by transmission electron microscopy (TEM) analysis. Their application performance, particularly in terms of foam properties, spreading behavior, and liquid crystal emulsion characteristics, was evaluated using dynamic foam analysis, dynamic contact angle measurements, and polarizing optical microscopy (POM). This study aims to provide fundamental data and practical insights to industrial applications.

## 2. Results

### 2.1. Structure Identification

**Figure 1:**
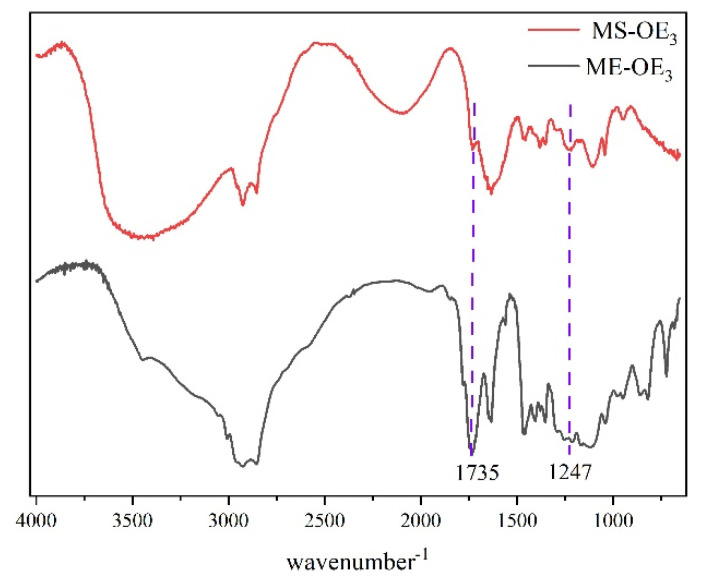
FT-IR spectra of ME-OE_3_ and MS-OE_3_.

The structures of the intermediate ME-OE_n_ (*n* = 3, 5, 7) and the final product MS-OE-OE_n_ (*n* = 3, 5, 7) were elucidated using FT-IR and ^1^H NMR spectroscopy. For illustrative purposes, ME-OE_3_ and MS-OE_3_ are discussed in detail. The FT-IR spectra of ME-OE_3_ and MS-OE_3_ are shown in Figure 1. The FT-IR spectrum of ME-OE_3_ displays a distinctive transmission decrement at approximately 1735 cm^−1^, corresponding to the carbonyl (C=O) stretch of the ester group. This peak is a clear indication of the successful formation of the monoester intermediate through the esterification process. The transmission decrement at approximately 1636 cm^−1^ is attributed to the stretching vibration of double bond (C=C). Upon examining the FT-IR spectrum of MS-OE_3_, a new transmission decrement emerges around 1247 cm^−1^. This peak is characteristic of the sulfonate (SO_3_^−^) group, signifying the successful incorporation of the sulfonate moiety into the molecule. Importantly, the ester carbonyl peak at 1735 cm^−1^ and the double bond peak at 1636 cm^−1^ are still evident in the spectrum of MS-OE_3_. This persistence of the transmission decrement indicates that the ester linkage and double bond in oleyl alcohol polyoxyethylene ether remain intact throughout the sulfonation process, preserving the structural integrity essential for the surfactant properties of the final product.

**Figure 2:**
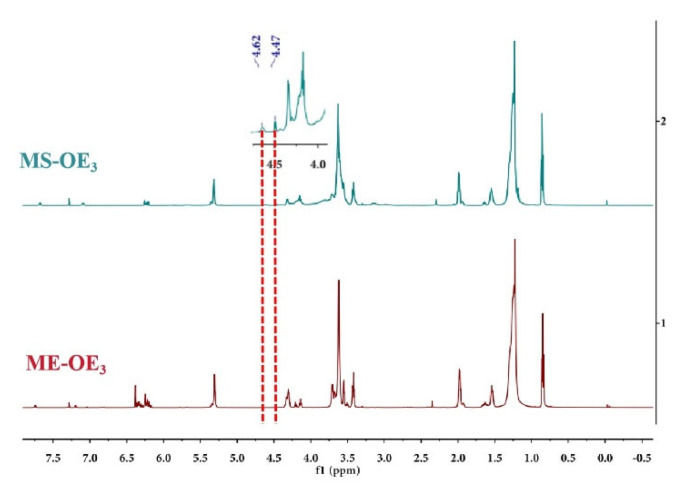
^1^HNMR spectra of ME-OE_3_ and MS-OE_3_.

The ¹H NMR spectra of ME-OE_3_ and MS-OE_3_ are presented in Figure 2. In the ¹H NMR spectrum of ME-OE_3_, the terminal methyl group (CH_3_) of the oleyl chain appears at δ 0.88 ppm, while the methylene protons (CH_2_) of the alkyl chains are observed at δ 1.20–1.61 ppm. The methylene protons adjacent to the olefinic protons (CH_2_-CH=CH) resonate in the range of δ 1.96–2.33 ppm, confirming the presence of the unsaturated alkyl chain. The methylene protons of the polyoxyethylene (OE) chain are detected at δ 3.40–3.69 ppm, verifying the OE group in the intermediate. The methylene protons adjacent to the ester linkage (O-CH_2_-CH_2_) appear at δ 4.19–4.28 ppm, indicating successful ester bond formation. Additionally, the olefinic protons (CH=CH) from the oleyl chain are observed at δ 5.28–5.33 ppm, further supporting the unsaturated structure. In the ¹H NMR spectrum of MS-OE_3_, all characteristic proton signals of ME-OE_3_ are retained, confirming that the core structure remains unchanged during sulfonation. New proton signals appear at δ 4.47–4.62 ppm, attributed to the methylene protons adjacent to the sulfonate group (SO_3_^−^). These signals confirm the successful introduction of the sulfonate moiety, indicating the completion of the sulfonation reaction. The combined FT-IR and ¹H NMR analyses provide conclusive evidence for the successful synthesis of the target oleyl-based sulfosuccinates, MS-OE_3_.

Detailed characterizations of MS-OE_5_ and MS-OE_7_ are provided in the Supporting Information, as shown in Figures S1 and S2.

### 2.2. Surface Activity

#### 2.2.1. Equilibrium Surface Tension

**Figure 3:**
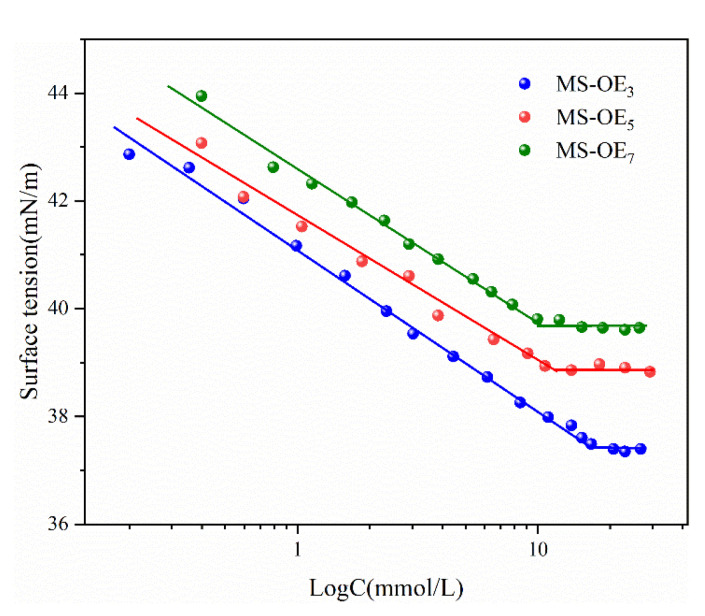
*γ*-logC curves of MS-OE_n_ in aqueous solutions at 25.0 °C.

The surface activity and adsorption behavior of MS-OE_n_ were thoroughly investigated through surface tension measurements. The relationship between surface tension (*γ*) and the logarithm of molar concentration (logC) was examined for MS-OE_n_ solutions, as depicted in Figure 3. The surface tension gradually decreased with increasing concentration until it reached a plateau, a characteristic behavior of surfactant solutions. The critical micelle concentration (cmc) and the corresponding surface tension at cmc (*γ*_cmc_) were determined from the intersection of two fitting lines (Table 1). The results indicate that the cmc decreases with an increase in the chain length of EO groups. This trend can be attributed to the enhanced hydrophilicity of the longer EO chains [14,15], which promotes micelle formation at lower concentrations. Additionally, the steric hindrance provided by the longer EO chains results in a less tight arrangement between molecules at the air/liquid interface, further facilitating micelle formation. The observed correlation between EO chain length and cmc provides insight into the aggregation behavior of MS-OE_n_. Specifically, the reduced steric hindrance in shorter EO chains (MS-OE_3_) may facilitate vesicle formation, as evidenced in Section 2.3.

Interestingly, while the cmc decreases with increasing EO units, the *γ*_cmc_ values show a slight increase. This observation suggests that the longer EO chains may introduce steric hindrance at the air/liquid interface, slightly reducing the efficiency of the surfactant in lowering the surface tension. This phenomenon is consistent with previous studies, which have reported that the presence of longer EO chains can lead to a less compact arrangement of surfactant molecules at the interface, thereby affecting their surface activity [16].

Furthermore, the influence of EO chain length on the cmc of MS-OE_n_ is significantly more pronounced than its effect on *γ*_cmc_. This indicates that while the addition of EO groups enhances the micellization process by reducing the cmc, it has a relatively minor impact on the equilibrium surface tension at the cmc. This finding underscores the importance of optimizing the EO chain length to achieve a balance between micelle formation and surface tension reduction, which is crucial for applications requiring precise control over surfactant behavior.

The surface excess concentration (*Γ*_max_) and the minimum area per molecule (*A*_min_) at air/liquid interface are determined using the Gibbs adsorption equation through Equations (1) and (2). Subsequently, the standard Gibbs free energy for both aggregation (Δ*G*_mic_) and adsorption (Δ*G*_ads_) can be derived from Equations (3) and (4) [17,18].

(1) $$\Gamma_{\max}=-\frac{1}{2.303nRT}(\frac{\partial \gamma }{\partial lgC})$$

(2) $$A_{min}=\frac{10^{16}}{N_{A}\Gamma_{max}}$$

(3) $${∆G}_{mic=}nRTln\left(\frac{cmc}{55.5}\right)$$

(4) $${∆G}_{ads}=nRTln\left(\frac{C_{\Pi }}{55.5}\right)-6.022\Pi A_{min}$$

where *n* = 3, R represents the gas constant, *T* is the absolute temperature, (*∂γ*/*∂lgC*) is the slope of the *γ*-logC curve before the cmc. Additionally, *N*_A_ denotes Avogadro’s number, *Π* (defined as *γ*_0_-*γ*) is the surface pressure at saturation, with *γ*_0_ being the surface tension of pure water, and C_Π_ corresponds to the surfactant’s molar concentration in the aqueous phase at surface pressure *Π*. All derived parameters are summarized in Table 1.

As can be seen in Table 1, the *A*_min_ values exhibit a gradual increase with the chain length of EO groups. Conversely, the *Γ*_max_ values show a decreasing trend. This behavior can be attributed to the coiling of the EO chains as their length increases, which enlarges the head group volume of the surfactant molecules. Consequently, fewer surfactant molecules can be accommodated at the interface upon reaching saturation, leading to a decrease in *Γ*_max_. However, since the differences in EO chain length among the MS-OE_n_ molecules are relatively small, the overall changes in both *A*_min_ and *Γ*_max_ are not significant. In addition, both Δ*G*_mic_ and Δ*G*_ads_ are negative, indicating that the adsorption and micellization processes of MS-OE_n_ in aqueous solution are spontaneous [19]. Furthermore, in all cases, the absolute value of Δ*G*_ads_ is significantly larger than that of Δ*G*_mic_, suggesting that the adsorption process at the air/liquid interface is more thermodynamically favorable compared to micellization. The values of both Δ*G*_mic_ and Δ*G*_ads_ for MS-OE_n_ become more negative with increasing EO chain length. This indicates that as the EO chain length increases, MS-OE_n_ molecules exhibit enhanced tendencies to adsorb at the interface and to form micelles in aqueous solutions [20,21].

#### 2.2.2. Dynamic Surface Tension

**Figure 4:**
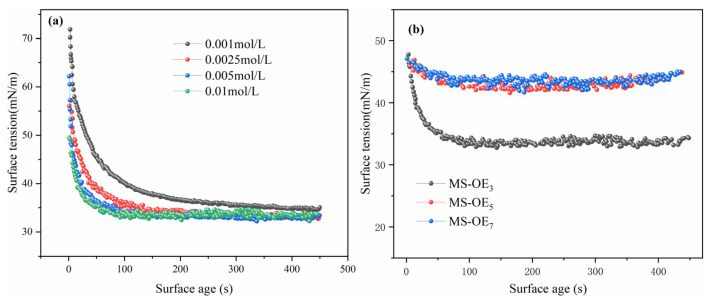
Dynamic surface tension as a function of surface age for (**a**) MS-OE_3_ at various concentrations and (**b**) MS-OE_n_ (*n* = 3, 5 7) at 0.01 mol/L.

The dynamic surface tension as a function of surface age for (a) MS-OE_3_ at various concentrations was shown in Figure 4a. The results indicate that surface tension steadily declines over time across all tested concentrations, eventually reaching a stable equilibrium state. At a concentration of 0.001 mol/L, the initial surface tension (near 0 s) is nearly identical to that of the pure solvent (72.0 mN/m for water). By approximately 250 s, the surface tension stabilizes and reaches equilibrium. As the surfactant concentration increases to 0.0025, 0.005, and 0.01 mol/L, the initial surface tension decreases more rapidly, and the time required to reach equilibrium is significantly reduced to around 120 s. This behavior can be attributed to the dynamic adsorption process, where new surfaces are continuously formed, and surfactant molecules migrate from the bulk phase to the air/liquid interface. The greater the concentration difference between the bulk phase and the adsorption layer, the faster the surfactant molecules migrate, resulting in a quicker reduction in surface tension and a shorter equilibrium time. This is consistent with reported results for both carbohydrate surfactants [22] and siloxane surfactants [23]. Additionally, the dynamic surface tension curves for MS-OE_3_ at concentrations of 0.0025, 0.005, and 0.01 mol/L are nearly identical, indicating that once the concentration exceeds 0.0025 mol/L, further increases in concentration have minimal impact on the dynamic surface tension. Furthermore, the equilibrium surface tension for MS-OE_3_ at different concentrations obtained from the dynamic surface tension measurements in Figure 4a stabilizes at approximately 35.0 mN/m, which is nearly consistent with the results obtained from the equilibrium surface tension measurements.

Figure 4b depicts the dynamic surface tension as a function of surface age for MS-OE_n_ (*n* = 3, 5 7) at 0.01 mol/L. It can be observed that the surface tension of MS-OE_3_ decreases rapidly from an initial value of 47.7 mN/m to 35.0 mN/m over time. Conversely, MS-OE_5_ and MS-OE_7_ display a smaller decrease in surface tension, from an initial value of 47.3 mN/m to 42.8 mN/m. This difference arises because the diffusion of surfactant molecules from the bulk phase to the interface is influenced not only by concentration but also by the steric hindrance of the molecules. The longer and more coiled EO chains in MS-OE_5_ and MS-OE_7_ introduce greater steric hindrance, resulting in slower diffusion rates.

### 2.3. Aggregation Behavior

**Figure 5:**
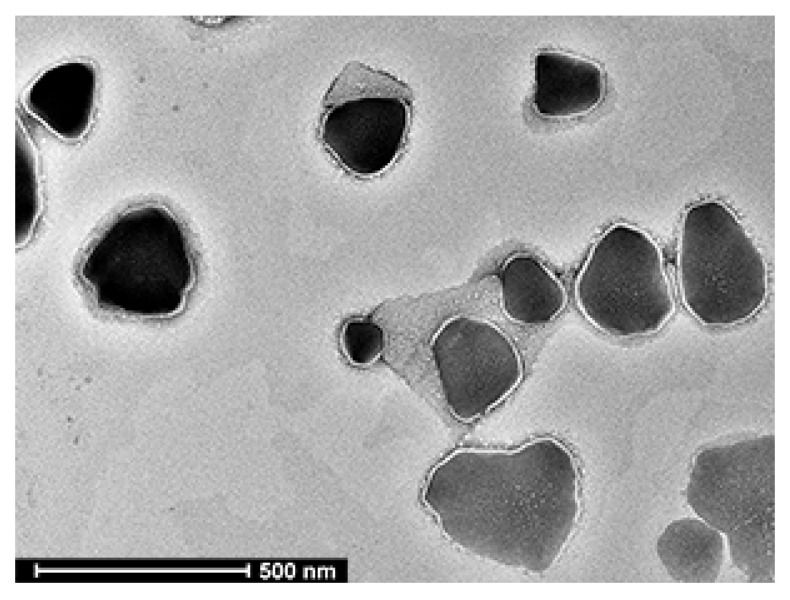
TEM images of negatively-stained aggregates formed in 0.1 mol/L MS-OE_3_ solutions.

Above cmc, the solutions of MS-OE_3_ exhibit turbidity, indicating the formation of large aggregates. To further elucidate the morphology of the aggregates in the MS-OE_n_ solutions, detailed analysis was conducted using negative-stain transmission electron microscopy (TEM). As illustrated in Figure 5, vesicles were distinctly observed in the 0.1 mol/L aqueous solution of MS-OE_3_. Conversely, in the MS-OE_5_ and MS-OE_7_ aqueous solutions at the same concentration, no such vesicles were detected. Considering the large hydrophilic headgroup of MS-OE_3_, spherical micelles were initially expected to form in aqueous solution. However, contrary to this expectation, MS-OE_3_ assembled into vesicles.

**Figure 6:**
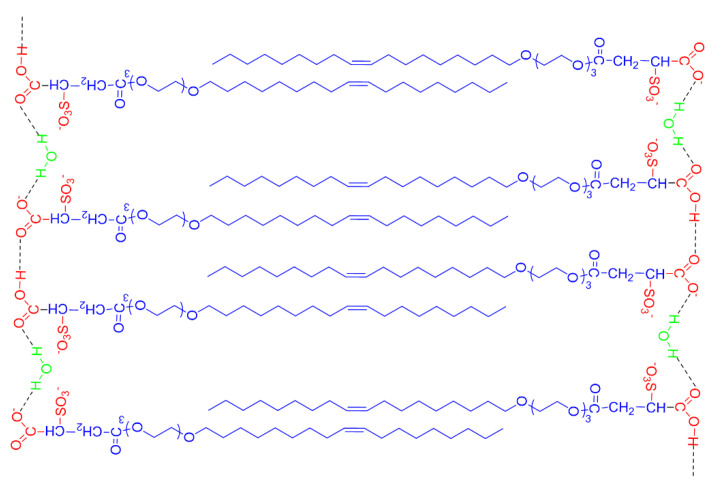
The schematic representation of bilayer formation of MS-OE_3_.

In general, double alkyl chains of amphiphiles, mimicking the structure of phospholipids, are prone to forming vesicle structures [24,25]. It has been reported that certain single-chain amphiphiles, such as derivatives of diphenylazomethine or biphenyl groups [26,27], are also capable of forming vesicles. Furthermore, single-chain surfactants can also assemble into vesicle structures through the combination of anionic and cationic surfactants [28,29,30], or pairing of long-chain carboxylic acids with salts [31,32,33]. However, there are relatively few studies on the vesicle formation of single-chain hydrocarbon-based amphiphiles without any additives. In our study, the single chain of a sulfosuccinate, which contains a carbonyl group, exhibits behavior akin to that of a dimer. This occurs through hydrogen bonding mediated by a water molecule between the carbonyl groups, as well as hydrogen bonding between carboxylic acid molecules. These interactions ultimately lead to the formation of a vesicle structure. The schematic representation of bilayer formation of MS-OE_3_ was shown in Figure 6. This phenomenon is reminiscent of the findings reported by K. Matsuoka on disodium lauryl sulfosuccinate [6] and by A. Roy on sodium 2-Dodecylnicotinate [34].

The aggregation behavior of surfactants in water depends on a delicate balance of forces acting between surfactant-surfactant and surfactant-water molecules [35]. In MS-OE_n_ solutions, the electrostatic repulsive force, hydrophobic interaction, van der Waals forces, and steric effect exist simultaneously among them. As the chain length of ethylene oxide (EO) groups in MS-OE_n_ molecules increases, the partially positive charge of the EO groups [36] reduces electrostatic repulsion, while hydrophobic interactions, van der Waals forces, and steric hindrance gradually intensify. The interplay of these forces collectively influences the aggregation behavior of MS-OE_n_.

### 2.4. Foaming Behavior

**Figure 7:**
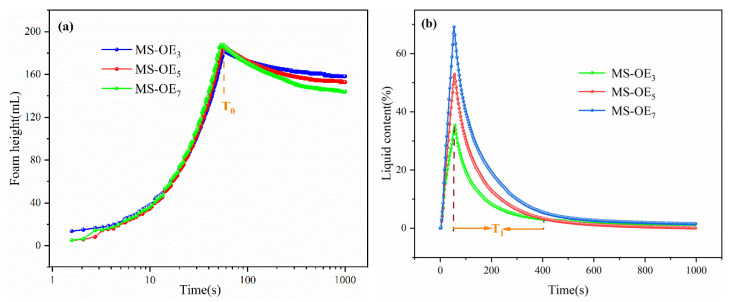
The foam height (**a**) and liquid content (**b**) of MS-OE_n_ aqueous solutions as a function of time at concentrations of 0.02 mol/L.

EO chain length in MS-OE_n_ tunes surfactant adsorption and interfacial properties, which collectively dictate performance in foam-related applications. Foamability and foam stability are two critical characteristics for evaluating the foaming performance of surfactants, which are crucial for their applications in various industries such as detergents, cosmetics, firefighting, and more. Figure 7 presents the foam height and liquid content of MS-OE_n_ aqueous solutions as a function of time at a concentration of 0.02 mol/L, measured under a constant gas flow rate of 150 mL/min.

In Figure 7a, T_0_ represents the time when gas flow is stopped, and the foam height at this point can be used to indicate the foamability of the surfactants. After T_0_, the foam begins to drain and decay. As illustrated in Figure 7a, when the foam height of the system reaches 180 mL, the foaming time for the MS-OE_n_ solutions is approximately 56 s, indicating comparable foamability among the three surfactants. This similarity in foamability is attributed to the equivalent capacities of the three surfactants to reduce water’s surface tension, as evidenced by their nearly identical *γ*_cmc_ values (Table 1). During foam formation, the expansion of the air/liquid interface increases the system’s surface free energy. However, surfactants with lower surface tension incur a smaller energetic penalty during this process, thereby enhancing foam formation efficiency. However, after the gas flow is stopped, the foam height of MS-OE_n_ gradually decreases without a plateau phase, suggesting that the foam not only undergoes drainage but also experiences collapse. Furthermore, the decay rates of the foams follow the order: MS-OE_7_ > MS-OE_5_ > MS-OE_3_. This trend indicates that as the chain length of EO groups in the molecule increases, the foam stability deteriorates.

To further analyze the reasons affecting the foam stability of MS-OE_n_ aqueous solutions, the liquid content of MS-OE_n_ foam was measured, as illustrated in Figure 7b. In the MS-OE_n_ aqueous solution, the drainage process persisted for 400 s or longer, indicating good foam stability. Furthermore, the liquid content of the foams followed the order MS-OE_7_ > MS-OE_5_ > MS-OE_3_, which may account for the formation of smaller and finer bubbles in the corresponding liquid solutions. It is also noteworthy that the time required for each surfactant to reach drainage equilibrium, denoted as T_1_ in Figure 7b, does not vary significantly with the increase in the chain length of EO groups. However, a gradual increasing trend is observed, with MS-OE_3_ < MS-OE_5_ < MS-OE_7_. The longer T_1_ corresponds to a slower drainage speed, which in turn enhances foam stability. This trend suggests that, from the perspective of drainage speed, as the chain length of EO groups in the molecule increases, the foam stability also increases. Paradoxically, this trend contradicts the observed stability decline in Figure 7a, implying competing stabilization mechanisms.

Generally, foam stability is not only determined by drainage speed but also the strength of the liquid film [37,38,39]. While extended EO chains decelerate drainage, they simultaneously compromise interfacial film strength. The coiled EO configurations reduce surface film viscoelasticity and disrupt molecular packing at air/liquid interfaces. This dual effect explains the overall stability reduction with increasing EO chain length: although prolonged T_1_ improves drainage resistance, the dominant factor remains the weakened interfacial film strength. Consequently, foam stability in MS-OE_n_ systems is primarily governed by interfacial robustness rather than drainage kinetics.

**Figure 8:**
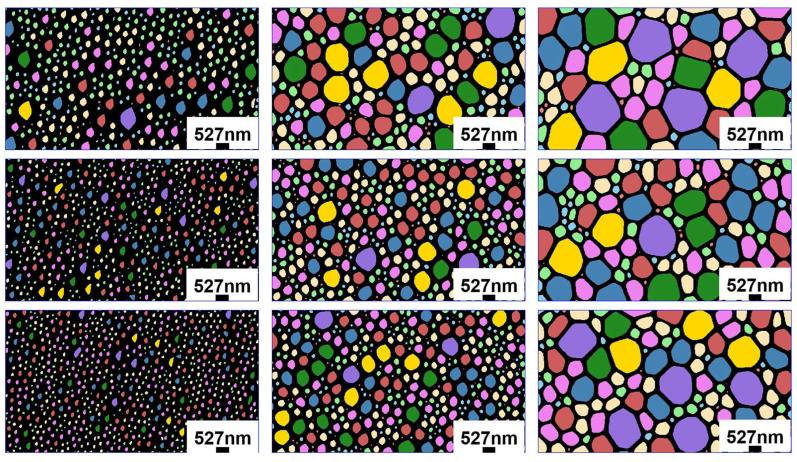
The bubbles’ structure of MS-OE_3_, MS-OE_5_ and MS-OE_7_ (from top to bottom) in aqueous solution at 1, 5, and 10 min (from left to right). The bars in the images are 527 nm.

Figure 8 provides a visual representation of the bubble structures formed by MS-OE_n_ in aqueous solutions at 1, 5, and 10 min. The corresponding bubble size distribution histograms for these surfactants at the same time intervals were presented in Figure S4. At 1 min, the bubbles of MS-OE_3_ solution are small, uniform, and densely packed, with sizes mainly around 167 μm. In the MS-OE_5_ solution, the bubbles formed are smaller than those in the MS-OE_3_ solution but still relatively uniform, with sizes mainly around 113 μm and a narrower distribution. The MS-OE_7_ solution, however, has a dense bubble structure with a predominance of very small bubbles, consistent with the higher liquid content shown in Figure 7b. The bubble size distribution histogram for MS-OE_7_ is centered around 102 μm, exhibiting the narrowest distribution range compared to other samples, indicating rapid bubble nucleation due to the longer EO chain length.

As time progresses, the bubbles gradually increase in size and transition into more regular structures. This evolution is driven by pressure differences between large and small bubbles during the foam decay process, a phenomenon known as Ostwald ripening [34]. In this process, gas diffuses from regions of higher pressure (smaller bubbles) to regions of lower pressure (larger bubbles), causing the larger bubbles to grow while the smaller bubbles shrink and eventually disappear. By 10 min, the bubble size for MS-OE_3_, MS-OE_5_, and MS-OE_7_ were mainly distributed at 475 μm, 413 μm, and 423 μm, respectively. Obviously, MS-OE_7_ exhibits rapid bubble growth and coalescence, indicating significant Ostwald ripening and coalescence, which result in poor foam stability. These observations align with the foam decay curves presented in Figure 7a, further confirming an inverse relationship between bubble stability and the chain length of EO groups within the molecular architecture.

### 2.5. Wetting Ability

Wettability holds significant importance in surfactant-based applications, especially in industries involving interactions with low-energy hydrophobic surfaces. Pesticide spraying, painting, and cosmetics are prime examples where this property plays a crucial role. In such applications, the efficacy of a surfactant solution is directly correlated to its ability to spread and adhere to these surfaces. By examining the time-dependent contact angle of MS-OE_n_ on a hydrophobic paraffin film, the wetting dynamics of MS-OE_n_ at the solid surface can be effectively analyzed.

**Figure 9:**
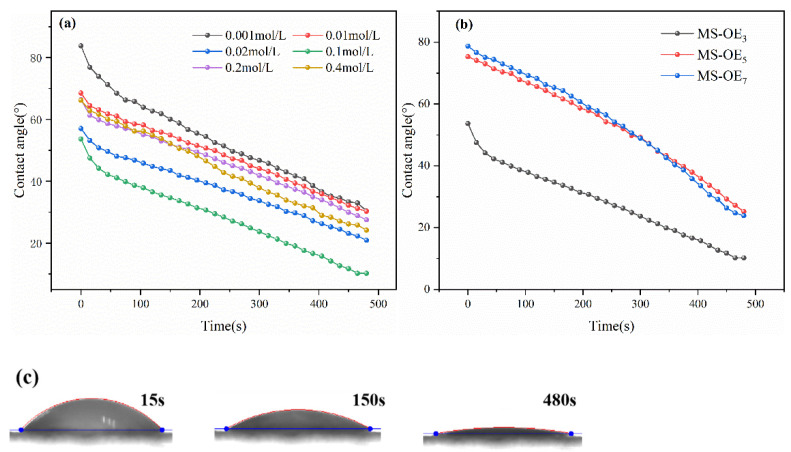
Dynamic contact angle of the surfactants as a function of time at 25 °C. (**a**) MS-OE_3_ with varying concentrations; (**b**) Different surfactants at 0.1 mol/L; (**c**) Contact angle images of 0.1 mol/L MS-OE_3_ at 15 s, 150 s and 480 s.

Figure 9a illustrates the dynamic contact angle of MS-OE_3_ solutions with varying concentrations at 25 °C over time. All tested concentrations demonstrated effective wetting behavior (θ < 90°), with the contact angle decreasing progressively over time. Below the cmc, the contact angle showed a concentration-dependent reduction, reaching a minimum value of 10.21° at 0.1 mol/L after 480 s. This optimal wetting performance can be explained by Young’s equation:

(5) $$\gamma_{sg}-\gamma_{sl}=\gamma_{lg}cos\;\theta$$

where *γ*_sg_, *γ*_sl_, and *γ*_lg_, are solid/gas interface, solid/liquid and liquid/gas interface tension, respectively. The *γ*_sg_ of the paraffin surface was measured to be 26 mN/m and remained constant regardless of surfactant concentration or time.

At sub-cmc concentrations, surfactant molecules adsorb at both the air/liquid interface, reducing *γ*_lg_, and the paraffin surface through hydrophobic interactions, with polar head groups oriented outward [40]. This dual adsorption mechanism simultaneously lowers *γ*_sl_ by enhancing solid/liquid interactions. The combined reduction of *γ*_lg_ and *γ*_sl_ drives the contact angle decrease, with higher concentrations (up to 0.1 mol/L) promoting greater interfacial adsorption and consequently lower contact angles.

Above the cmc, the air/liquid interface becomes saturated, stabilizing *γ*_lg_. However, surfactant molecules continue to adsorb at the solid/liquid interface, eventually forming a bilayer structure with hydrophobic tails oriented outward. This structural reorganization increases *γ*_sl_, leading to the observed contact angle increase to 27.52° and 24.14° at 0.2 mol/L and 0.4 mol/L, respectively. The concentration-dependent transition from monolayer to bilayer adsorption at the solid/liquid interface provides a comprehensive explanation for the non-monotonic wetting behavior observed across different surfactant concentrations.

Figure 9b shows the dynamic contact angles of MS-OE_n_ (*n* = 3, 5, 7) solutions at a concentration of 0.1 mol/L. As evident from the plot, MS-OE_3_ exhibits the lowest contact angle at any given spreading time, while MS-OE_5_ and MS-OE_7_ maintain consistently higher and nearly identical contact angles. The coiled EO chains in MS-OE_5_ and MS-OE_7_ molecules create significant steric hindrance, which reduces the number of molecules that can adsorb at the air/liquid and solid/liquid interfaces. Consequently, the reduction in both *γ*_lg_ and *γ*_sl_ is less pronounced, resulting in larger contact angles compared to MS-OE_3_. Contact angle images of 0.01 mol/L MS-OE_3_ at 15 s, 150 s and 480 s can be obtained in the Figure 9c.

### 2.6. Liquid Crystal Emulsion

Liquid crystals (LCs) represent a unique state of matter that exhibits both the molecular order of crystals and the fluidity of liquids, making them particularly valuable in cosmetics, drug delivery systems, and advanced functional materials [41]. The stability assessment of emulsions formulated with MS-OE_n_ surfactants revealed significant differences in their performance over time. The emulsion containing MS-OE_3_ underwent phase separation within 13 days after preparation. In contrast, the system containing MS-OE_5_ maintained its structural integrity for 50 days prior to showing signs of delamination. Notably, the formulation incorporating MS-OE_7_ demonstrated remarkable long-term stability, with no observable macroscopic phase separation even after 90 days.

**Figure 10:**
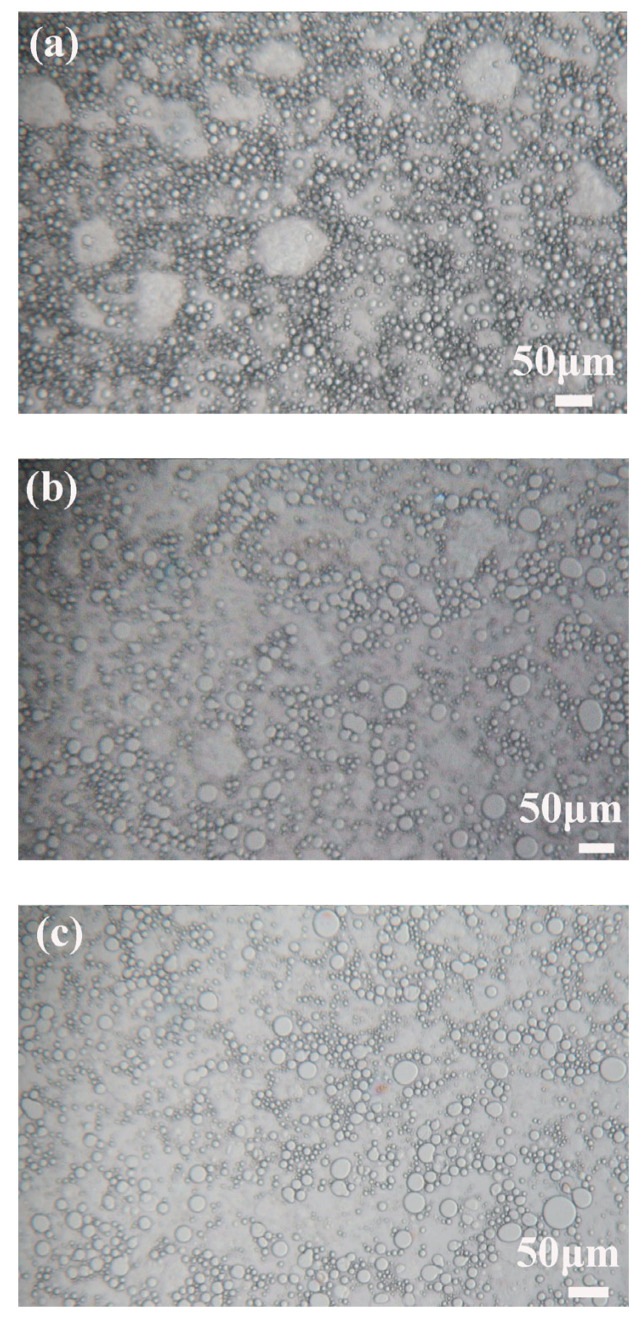
The droplet morphology of the emulsions. (**a**) MS-OE_3_; (**b**) MS-OE_5_; (**c**) MS-OE_7_.

Two days after the preparation of the emulsions, the droplet morphology was analyzed. As shown in Figure 10, the emulsion droplets formed using MS-OE_n_ as the surfactant exhibited a spherical shape. Notably, the droplet size distribution became broader as the value of *n* in MS-OE_n_ increased. Despite this variation, the average particle size of all three emulsions was measured to be around 9.55 nm using Nano Measurer software.

**Figure 11:**
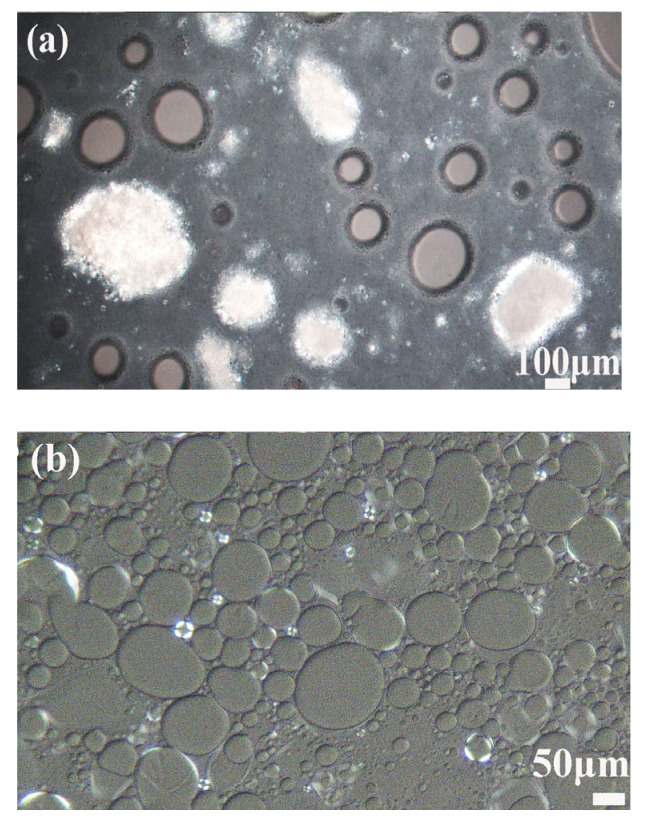
Structural textures of liquid crystal emulsion under POM. (**a**) MS-OE_5_; (**b**) MS-OE_7_.

Subsequent POM analysis indicated that both the emulsion formulated with MS-OE_5_ and MS-OE_7_ formed birefringent liquid crystalline textures featuring the characteristic oily texture and Maltese cross patterns, respectively (Figure 11). This suggests the presence of ordered lamellar arrangements. However, nothing can be found in the emulsion formulated with MS-OE_3_. The interfacial layer in systems capable of forming liquid crystals exhibits viscoelastic properties. This viscoelasticity imparts mechanical resilience to the interfacial film, rendering it resistant to rupture under external stress and enabling rapid self-repair upon damage. Consequently, such systems demonstrate superior emulsion stability.

## 3. Materials and Methods

### 3.1. Materials

OE_n_ *n* = 3, 5, 7 (Industry Grade) was purchased from Zhejiang Kaide Chemical Co., LTD. MAH (Hangzhou, China) and sodium sulphite were provided by Shanghai Aladdin Biochemical Technology Co., Ltd. (Shanghai, China). p-Toluenesulfonic acid were supplied by Shanghai Lingfeng Chemical Reagent Co., Ltd. (Shanghai, China). Liquid paraffin was offered by Tianjin Komio Chemical Reagent Co., Ltd. (Tianjin, China). All reagents were analytical grade and without further purification, unless otherwise stated. Ultrapure water with a resistivity 18.25 MΩ cm was used.

### 3.2. Synthesis of MS-OE_n_

In a four-necked flask equipped with a condenser, electric stirrer, and thermometer, OE_n_ and MAH were added in a molar ratio of n_OEn_:n_MAH_ = 1:1.05, along with 1.5 wt% of p-toluenesulfonic acid as a catalyst based on the total mass. The reaction mixture was heated to 100 °C under a nitrogen atmosphere and stirred continuously for 2 h, producing a yellow-colored intermediate monoester of oleyl alcohol and maleic acid (ME-OE_n_). A 15% (wt%) aqueous solution of Na_2_SO_3_ was added to the intermediate product in a molar ratio of n_OEn_:n_Na2SO3_ = 1:1.01. The mixture was then heated to 100 °C and stirred for 2 h. This resulted in the formation of a yellow product (MS-OE_n_).

The product obtained above was neutralized to pH 7–8 with a 4% NaOH solution. Ethanol was then added, and inorganic salts were removed by hot filtration. After ethanol evaporation, petroleum ether extraction was performed to separate unreacted oil, followed by rotary evaporation of the aqueous phase to obtain a viscous yellow product. The purified MS-OE_3_, MS-OE_5_, and MS-OE_7_ exhibited active substance contents of 91.1%, 92.7%, and 95.1%, respectively.

### 3.3. Preparation of Emulsion

The emulsion was prepared using an emulsifier blend of cetearyl alcohol and surfactant MS-OE_n_ in a 6:4 mass ratio. In the process, 10% of this emulsifier mixture, 20% liquid paraffin, and 70% ultrapure water were mixed and heated to 80.0 °C while being mechanically stirred at 10,000 rpm for 5 min. After cooling to room temperature, a homogeneous, creamy emulsion was obtained.

### 3.4. Characterization

#### 3.4.1. Structural Characterization

The structural characterization of MS-OE_n_ was performed using Fourier transform infrared (FT-IR) spectroscopy (Nicolet iS20, Thermo Fisher Scientific, Waltham, MA, USA) and nuclear magnetic resonance (¹H NMR) spectroscopy (Quantum-I Plus 600 M, Bruker, Rheinstetten, Germany). The ¹H NMR chemical shifts were calibrated relative to the CDCl_3_ signal at δ 7.26 ppm.

#### 3.4.2. Surface Tension Measurement

The equilibrium surface tension was determined with a Krüss K12 tensiometer (Krüss Company, Hamburg, Germany) using the single-measurement technique at 25.0 °C. The dynamic surface tension measurements were conducted at 25.0 °C using the pendant drop method with a DSA25R instrument (Krüss GmbH, Hamburg, Germany). Freshly prepared solutions were aged for at least 24 h before the surface tension measurements were conducted. The double-distilled water used as a reference had a surface tension of 72.0 ± 0.2 mN/m.

#### 3.4.3. Transmission Electron Microscopy (TEM)

The morphological characteristics of MS-OE_n_ aggregates in solution were investigated utilizing a Talos L120C (Thermo scientific, Waltham, MA, USA) transmission electron microscope operating at an accelerating potential of 120 kV. Specimens for TEM analysis were prepared by depositing MS-OE_n_ drops onto carbon-supported copper grids, followed by negative staining with 2% (wt%) phosphotungstic acid solution. The resulting microstructural images of the aggregates were subsequently captured for detailed examination.

#### 3.4.4. Foam Properties Measurement

The foamability and foam stability of MS-OE_n_ were evaluated using a DFA100 dynamic foam analyzer (Krüss Company, Hamburg, Germany) at 25.0 °C. A 0.02 mol/L surfactant solution was transferred into the measuring cylinder of the instrument, ensuring no air bubbles were introduced during the process. Foam generation was initiated by introducing sparging air at a constant flow rate of 150 mL/min in a glass column (35 mm in inner diameter) using 60 mL surfactant solutions, and the process was halted once the foam reached a predetermined height of 180 mm. The bubble size and size distribution of MS-OE_n_ foams in aqueous solution were analyzed using the Foam scan (DSA100) equipped with a foam structure module. Each measurement was performed in triplicate to ensure reproducibility, and the average values were reported.

#### 3.4.5. Wetting Ability Measurement

The spreading dynamics and contact angles of MS-OE_n_ aqueous solutions were assessed on hydrophobic surfaces using a Krüss DSA 25 goniometer (Krüss GmbH, Germany). Paraffin was employed as hydrophobic substrates to investigate the wettability characteristics at 25.0 °C. To achieve high measurement accuracy, each sample was tested at least three times. The reported contact angles represent the mean values obtained from these replicate measurements.

#### 3.4.6. Polarizing Microscope (POM) Measurement

The liquid crystal phase structures of the emulsion were investigated using an LxPOL POM (LaboAmerica, Fermont, CA, USA) at 25.0 °C. Samples were prepared by sandwiching a small amount of the emulsion between a glass slide and a coverslip. And then the slide was observed with polarizing microscope under bright field and polarized light. The bright field image was used to analyze the droplet size and the polarized light image was employed to observe the textures and birefringence patterns.

## 4. Conclusions

In this study, three oleyl-based sulfosuccinates (MS-OE_n_, *n* = 3, 5, 7) with varying EO chain length were successfully synthesized and characterized. The interplay between EO chain length and surfactant architecture dictates performance: Shorter chains (MS-OE_3_) prioritize interfacial adsorption and promote vesicle formation, conferring superior foam stability and exceptional wetting properties (θ = 10.21°). Conversely, longer chains (MS-OE_5_/_7_) enhance micellar packing, trading off wetting efficiency in favor of liquid crystal emulsion formation and stabilization. This trade-off underscores the critical role of EO chain modulation in tailoring surfactant function for specific applications.

## Figures and Tables

**Table 1:** Parameters of surface properties for MS-OE_n_ aqueous solutions.

MS-OE*_n_*	cmc	*γ*_cmc_	*Γ*_max_	*A*_min_	Δ*G*_mic_	Δ*G*_ads_
	mmol/L	mN/m	μmol/m^2^	nm^2^	KJ/mol	KJ/mol
MS-OE_3_	17.7	37.4	0.16	10.08	−11.39	−291.14
MS-OE_5_	11.4	38.8	0.15	11.10	−15.82	−311.58
MS-OE_7_	9.9	39.6	0.14	12.10	−17.23	−331.75

## Data Availability

Data is contained within the article.

## Supplementary Materials

The following supporting information can be downloaded at: https://www.mdpi.com/article/10.3390/molecules30112321/s1, Figure S1: FT-IR spectra of ME-OE_5_ and MS-OE_5_ (a)_,_ and ME-OE_7_ and MS-OE_7_(b); Figure S2: ^1^HNMR spectra of ME-OE_5_ and MS-OE_5_ (a)_,_ and ME-OE_7_ and MS-OE_7_ (b); Figure S3: The bubble size distribution of MS-OE_3_, MS-OE_5_ and MS-OE_7_ (from top to bottom) in aqueous solution at 1, 5, and 10min (from left to right).
