# Supplementary material for: Polyoxyethylene Group-Dependent Surface Properties and Aggregation Behavior in Oleyl-Based Sulfosuccinate Systems
Source: Molecules. 2025 May 26;30(11):2321. doi: 10.3390/molecules30112321 (PMC12156318; doi:10.3390/molecules30112321)
Supplement: Supplementary file 1 [file molecules-30-02321-s001.zip › molecules-3612295-supplementary.pdf]

Supporting information

Polyoxyethylene Group-Dependent Surface Properties and Aggregation Behavior in Oleyl-Based Sulfosuccinate Systems

Ping Li*, Zhengwei Zhang, Jie Chai, Yuan Liu, Siqi Han, Peixin Bai

High Value Fine Chemicals Research Center, Department of Chemistry and Chemical Engineering, Jinzhong University, Jinzhong Shanxi, 030619, P. R. China

*Corresponding Author

Address: Department of Chemistry and Chemical Engineering, Jinzhong University, Jinzhong Shanxi, 030619, P. R. China

Tel: +8613485350814; E-mail: yipingli_@126.com

Identification of the products

FT-IR

The Fourier-transform infrared (FT-IR) spectra of ME-OE_5 and MS-OE_5, as well as ME-OE_7 and MS-OE_7, are depicted in Figure S1(a) and (b), respectively. For ME-OE_5 and ME-OE_7, a prominent absorption peak at approximately 1735 cm^{-1} is observed, which is attributed to the carbonyl (C=O) stretching vibration of the ester functional group. This characteristic peak confirms the successful synthesis of the monoester intermediate via the esterification reaction. In contrast, the FT-IR spectra of MS-OE_5 and MS-OE_7 reveal an additional absorption peak at around 1247 cm^{-1}. This peak is indicative of the sulfonate (SO_3^-) group, thereby confirming the successful introduction of the sulfonate moiety into the molecular structure. Notably, the ester carbonyl peak at 1735 cm^{-1} is retained in the spectra of MS-OE_5 and MS-OE_7. The presence of this peak suggests that the ester linkage is preserved during the sulfonation process, which is crucial for maintaining the structural integrity required for the surfactant functionality of the final compounds.

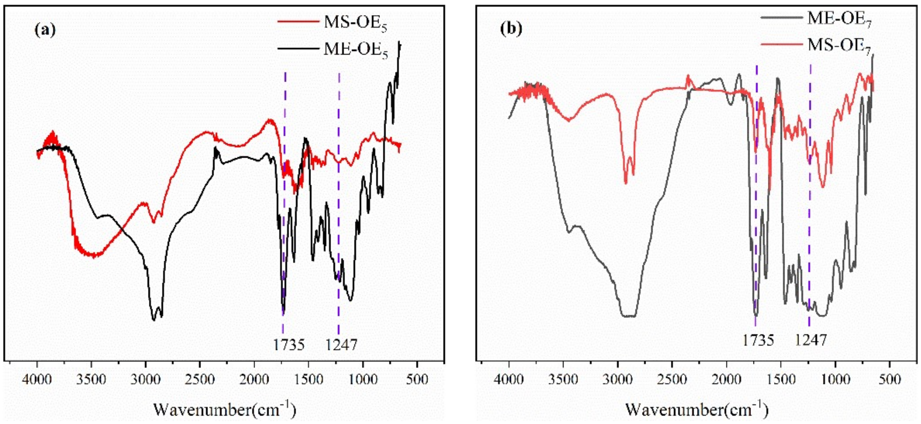

Figure S1. FT-IR spectra of ME-OE_5 and MS-OE_5 (a), and ME-OE_7 and MS-OE_7(b).

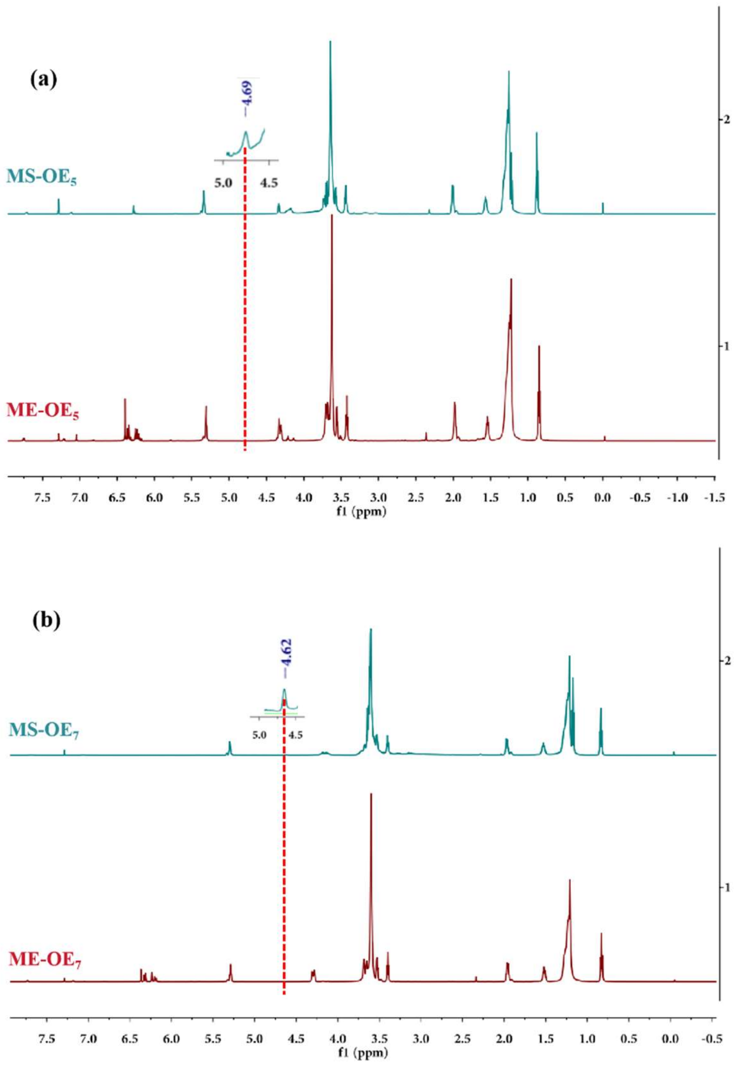

Figure S2. ^{1}HNMR spectra of ME-OE_5 and MS-OE_5 (a), and ME-OE_7 and MS-OE_7(b).

^{1}HNMR spectra of ME-OE_5 and MS-OE_5, as well as ME-OE_7 and MS-OE_7, were depicted in Figure S2(a) and (b). Consistent with MS-OE_3 (in Figure 2), in the ^{1}HNMR spectra of MS-OE_5 and MS-OE_7, all characteristic proton signals of ME-OE_5 and ME-OE_7 are retained, confirming that the core structure remains unchanged during sulfonation. New proton signals appear at δ 4.69 ppm and δ4.29 ppm, attributed to the methylene protons adjacent to the sulfonate group (SO_3^-) for MS-OE_5 and MS-OE_7, respectively. These signals confirm the successful introduction of the sulfonate moiety,

indicating the completion of the sulfonation reaction. The combined FT-IR and ^{1}H NMR analyses provide conclusive evidence for the successful synthesis of the target oleyl-based sulfosuccinates, MS-OE_5 and MS-OE_7.

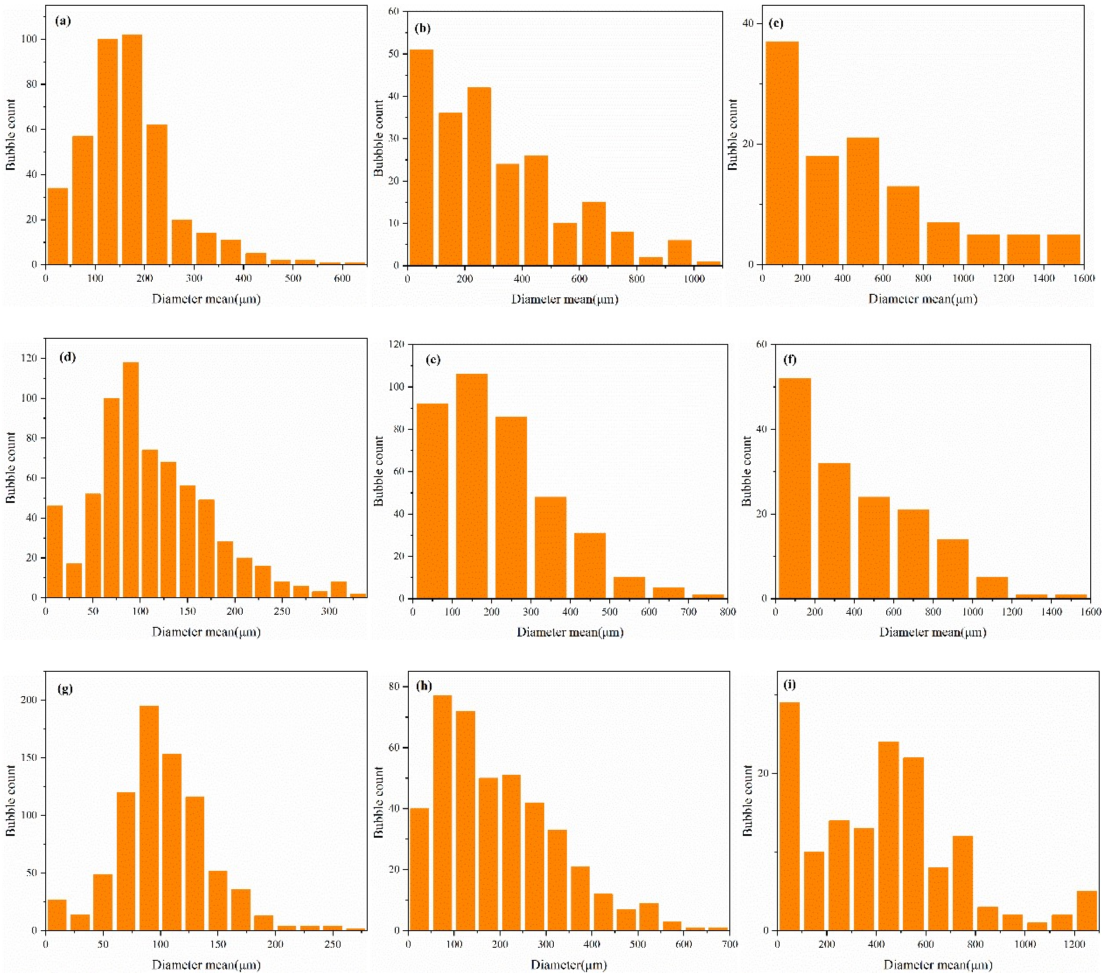

Figure S3. The bubble size distribution of MS-OE_3, MS-OE_5 and MS-OE_7 (from top to bottom) in aqueous solution at 1, 5, and 10min (from left to right).
